# Eliminating Explicit and Implicit Biases in Health Care: Evidence and Research Needs

**DOI:** 10.1146/annurev-publhealth-052620-103528

**Published:** 2022-01-12

**Authors:** Monica B. Vela, Amarachi I. Erondu, Nichole A. Smith, Monica E. Peek, James N. Woodruff, Marshall H. Chin

**Affiliations:** 1 Department of Medicine, Section of Academic Internal Medicine, University of Illinois College of Medicine in Chicago, Chicago, Illinois, USA; 2 Department of Internal Medicine and Pediatrics, University of California, Los Angeles Medical Center, Los Angeles, California, USA; 3 Department of Internal Medicine, Hospital of the University of Pennsylvania, Philadelphia, Pennsylvania, USA; 4 Department of Medicine, Section of General Internal Medicine and Chicago Center for Diabetes Translation Research, University of Chicago, Chicago, Illinois, USA; 5 Pritzker School of Medicine, University of Chicago, Chicago, Illinois, USA; 6 Department of Medicine and Chicago Center for Diabetes Translation Research, University of Chicago, Chicago, Illinois, USA

**Keywords:** bias, equity, disparity, racism

## Abstract

Health care providers hold negative explicit and implicit biases against marginalized groups of people such as racial and ethnic minoritized populations. These biases permeate the health care system and affect patients via patient–clinician communication, clinical decision making, and institutionalized practices. Addressing bias remains a fundamental professional responsibility of those accountable for the health and wellness of our populations. Current interventions include instruction on the existence and harmful role of bias in perpetuating health disparities, as well as skills training for the management of bias. These interventions can raise awareness of provider bias and engage health care providers in establishing egalitarian goals for care delivery, but these changes are not sustained, and the interventions have not demonstrated change in behavior in the clinical or learning environment. Unfortunately, the efficacy of these interventions may be hampered by health care providers’ work and learning environments, which are rife with discriminatory practices that sustain the very biases US health care professions are seeking to diminish. We offer a conceptual model demonstrating that provider-level implicit bias interventions should be accompanied by interventions that systemically change structures inside and outside the health care system if the country is to succeed in influencing biases and reducing health inequities.

## 1. INTRODUCTION

Although expressions of explicit bias have declined in the United States over time, implicit bias has remained unrelenting. Health care providers hold negative explicit and implicit biases against many marginalized groups of people, including racial and ethnic minoritized populations, disabled populations, and gender and sexual minorities, among others (29, 63). Implicit bias permeates the health care system and affects patients via patient–clinician communication, clinical decision making, and institutionalized practices (78). Higher education systems, including medical schools and academic hospitals, have been affected by the discrimination and bias that have long permeated the health care delivery system (84, 104). Bias in admissions and promotions processes, in classroom and bedside instruction, and by health care providers contributes to the constant messaging that stereotypes and isolates marginalized groups (80, 102, 105). These biases hinder improvement in compositional diversity of health care providers, long recognized as an important mechanism in reducing health care disparities (60). This complex system of discrimination and biases causes devastating health inequities that persist despite a growing understanding of the root causes and the health care system’s professional, ethical, and moral responsibility to address these inequities.

It has been theorized that implicit bias and structural racism mutually reinforce one another—ambient structural racism and its outcomes reinforce an individual’s psychological associations between racial identity and poorer outcomes (implicit bias) (20, 21). Inequitable structural determinants have diminished housing, education, health care, and income and have increased exposure to environmental pollutants and chronic stressors for marginalized populations (76, 108). Structural inequities and discrimination have created stereotypes of marginalized populations or communities and implicit and explicit biases toward them. Health care providers hold negative explicit and implicit biases against racialized minorities. A similar reinforcing dynamic may exist for marginalized populations such as those who are overweight/obese, use wheelchairs, have limited English proficiency, have mental health illness, and belong to lower socioeconomic classes (29). These biases can facilitate the creation and perpetuation of discriminatory systems and practices, creating a complex feedback loop that sustains itself.

Addressing bias remains a fundamental professional responsibility of health care and public health professionals accountable for population health and wellness (64, 65). This article (*a*) provides an overview of existing evidence of bias among health professionals, health practitioners, and public health workers in the practice and training environments (and lay health workers as appropriate) and its impact on health disparities; (*b*) systematically reviews the extant literature for evidence and limitations of current interventions designed to reduce or manage biases; (*c*) explores the interaction between bias and structural elements of the health care system (including medical education); and (*d*) proposes a conceptual model that frames bias not as an independent factor in the generation of disparities but as one element of a reinforcing system of elements that perpetuates such disparities. Ultimately, we provide evidence that interventions designed to reduce or manage existing explicit and implicit biases in clinical settings and public health are insufficient and will continue to fall short in reducing health inequities if we do not concomitantly address the racism and discrimination ingrained in health, medical educational systems, and other societal structures.

## 2. BACKGROUND

### 2.1. Overview of Bias

Critical to an understanding of interventions that address explicit and implicit biases in health care is an understanding of key terminology, tools used to measure bias, and the evidence for and impact of these biases in health care.

#### 2.1.1. Key terminology: What are implicit and explicit biases?

Implicit biases are unconscious mental processes that lead to associations and reactions that are automatic and without intention; actors have no awareness of the associations with a stimulus (41, 43) (Table 1). Axt et al. (4) maintain that social status is relational and people unconsciously hold more negative attitudes or feelings about membership of an outgroup (people with whom they do not share identities) than about membership of an ingroup (people with whom they share identities). A stereotype is a fixed set of attributes associated with a social group (49).

Implicit bias goes beyond stereotyping to include favorable or unfavorable evaluations toward groups of people (Table 1). Although we are not aware these implicit biases exist, they have a significant impact on decision making (97).

A belief is explicit if consciously endorsed (43). Explicit forms of bias include preferences, beliefs, and attitudes of which people are generally consciously aware, personally endorse, and can identify and communicate (22). Discrimination, the result of either implicit or explicit biases, is the inequitable treatment and/or impact of general policies, practices, and norms on individuals and communities based on social group membership (65, 76). Daumeyer et al. (22) argue that implicit biases must be exposed and discussed so that people and institutions can be held accountable for their effects. They argue for nuanced conversations about the ways in which implicit biases shape behavior and the ways to combat it.

#### 2.1.2. Tools used to measure implicit bias: How good are these measures? Have they been used outside of medicine?

In 1998, Greenwald et al. (45) described a word association test that identified implicit stereotype effects through indirect reaction time measures even when subjects self-reported low measures of prejudice. Since then, the implicit association test (IAT) has consistently demonstrated implicit stereotyping for a range of different social categories, particularly gender and ethnicity (Table 1). Greenwald et al. (42) maintain that statistically small effects of the IAT can have socially large effects. A meta-analysis by Greenwald et al. (45) demonstrated the predictive validity of the IAT regarding implicit stereotype associations to behavioral outcomes across a range of social subject areas. Some critics challenge whether the IAT measures implicit bias and predicts behavior, and question its utility in clinical and other real-world situations (3, 69). Most researchers agree that the IAT has limitations (44). It does not have high test-retest reliability in the same individual, and it is not useful as a tool to label individuals as implicitly sexist or racist or to predict behavior (73). The IAT has been used in health professions education as a metric to demonstrate the efficacy of educational interventions meant to reduce implicit bias and as a tool to raise awareness of existing implicit bias among health care trainees and providers (101).

#### 2.1.3. Implicit biases in health care: What is the evidence for racial bias among health care professionals? What is the impact of such bias in health care?

Implicit racial and ethnic bias exists among health care professionals in favor of White patients and against Black, Hispanic, and dark-skinned patients even when all other major factors (e.g., socioeconomic differences, insurance status) have been controlled and accounted for. Hall et al. (47) published a systematic literature review of 15 studies designed to explore the evidence of provider implicit racial bias and health outcomes. In the studies measuring prevalence, rates of anti-Black bias in health care providers ranged from 42% to 100%. These findings were redemonstrated in similar reviews conducted in 2017 (29) and 2018 (63).

Hoffman et al. (50) demonstrated in 2016 that White medical students and residents were more likely to believe that Black patients had thicker skin and smaller brains, and were more likely to rate Black patients as feeling less pain than and not needing the same levels of pain medications as White patients. Several studies have demonstrated that negative implicit biases held by those in the health professions are similar to those seen in the lay population (29).

The Medical Student Cognitive Habits and Growth Evaluation Study (CHANGES) has provided the greatest insight into the implicit and explicit biases held by medical students and trainees in the United States. This longitudinal multimeasure study followed a large sample of students attending a stratified random sample of 49 US allopathic medical schools and measured associations between possible interventions and levels of biases held by students. A web-based survey completed by more than 4,500 first-year medical students demonstrated that most students exhibited implicit (74%) and explicit (67%) weight bias. The study also demonstrated that scores of implicit weight bias were similar to scores of implicit bias against racial minorities (74%) in the same group of students (86). The size and scope of this study demonstrate undeniable evidence that implicit bias is pervasive among medical students, even in the first year of medical school. The multiple papers and findings generated by this foundational study were excluded from the final selection of studies in the results section because the study was observational and did not introduce interventions.

Biases affect health care delivery and public health outcomes, the health professions workplace and learning environments, and the diversity of trainees and workforce (Table 2). Hall et al. (47) demonstrated that these implicit biases have negatively affected patient–provider interactions, treatment decisions, and patient adherence to treatment. The most consistent evidence is found in studies of patient–provider interactions in which the bias of health care providers has been repeatedly linked to discriminatory care (18)—patients rate physicians with higher levels of implicit bias as less patient-centered in the primary care setting. Blanchard & Lurie (6) demonstrated that patients who perceived that they would have received better treatment if they were of a different race were significantly less likely to receive optimal chronic disease screening and more likely to not follow the doctor’s advice or to delay care. In a large study of adult primary care, higher implicit bias among health care providers was associated with patients’ lower ratings of interpersonal treatment, contextual knowledge, communication, and trust (5).

Other studies have confirmed associations between provider bias (demonstrated via IAT testing) and disparate treatment of their patients (63). In a systematic literature review, six studies found that higher implicit bias among health care providers was associated with disparities in treatment recommendations, expectations of therapeutic bonds, pain management, and empathy (63). Seven studies that examined the impact of implicit provider bias on real-world patient–provider interaction found that health care providers with stronger implicit bias demonstrated poorer patient–provider communication and that health care providers with high implicit biases (*a*) provided lower rates of postoperative narcotic prescriptions for Black children than for White children (93), (*b*) had poorer bonding with Black patients than with White patients (55), and (*c*) made disparate recommendations for thrombolytic therapy for Black patients and White patients (40).

A study of 3,756 students at 49 US medical schools demonstrated that high scores of racism as measured by the three variables were significantly correlated with low scores of student intentions to work in underserved areas and to provide care to minority populations (74).

Implicit bias affects not only patients but also trainees and faculty within health care systems. A 2014 systematic literature review revealed that rates of harassment and discrimination against trainees (24% reported racial discrimination, 33% reported sexual harassment, and 54% reported gender discrimination) have remained unchanged over time (31). Minority trainees report facing daily bias and microaggressions and having feelings of isolation and substantial stress (74). Minority medical students reported five-times-higher odds of racial discrimination and isolation than did nonminority peers (26). Stereotype threat (defined in Table 1) is common, particularly among non-White students, interferes with learning, and adds to the cognitive load of minoritized students (9). Thus, bias in health professions training can affect the performance of racialized minorities. Early and small differences in assessed clinical performance, which may be affected by implicit biases, lead to larger differences in grades and selection for awards [e.g., Alpha Omega Alpha Honor Medical Society (AOA)], ultimately affecting career trajectories of racial minority candidates (102). For example, significant differences in negative descriptive words on medical students’ evaluations have been found across different racial and gender groups (91). Membership in AOA, conferred to only 16% of each graduating medical school class, has effectively barred diversity in many specialties and may represent a longstanding form of structural racism (7).

### 2.2. Impact of Interventions Designed to Reduce or Manage Bias

Literature outside of health care has introduced techniques to manage implicit bias, including stereotype replacement (replacing stereotypical responses to bias with nonstereotypical ones), counter-stereotypic imaging (imagining known counter-stereotypical people), individuation (learning personal attributes of persons present rather than identifying group attributes), perspective taking (taking the perspective of persons present), and increasing opportunities for contact. Several studies have explored the efficacy of these interventions. Strikingly, the only study demonstrating reduction of measured implicit bias was conducted on undergraduate students enrolled in a course using a prejudice-habit-breaking intervention involving instruction of all the aforementioned techniques with effects lasting 8 weeks (24). Unfortunately, these results may not be generalizable and have not been reproduced. Lai et al. (57) tested nine interventions and although all immediately reduced implicit preferences, results were sustained for only several hours to days. FitzGerald et al. (30) conducted in 2019 a systematic review of bias interventions utilizing the IAT or other measures across multiple disciplines. They found that most studies did not provide robust data to support many interventions, although perspective taking was more successful than counter-stereotypic imaging.

### 2.3. Interactions Between Bias and Structural Elements of the Health Care System

Implicit bias has important interactions with structural elements of the health care system. Evidence suggests that implicit bias can reinforce structural dimensions of the health care system that generate disparities. Other evidence suggests that structural dimensions of the health care system and medical education can reinforce implicit bias. These interactions suggest a complex and mutually reinforcing relationship between implicit bias and structural elements of the health care system.

#### 2.3.1. The relationship between implicit bias and public policy.

Implicit biases influence the decisions of policy makers in government and health care that result in structural racism (70, 75, 81). Public health responses to the coronavirus disease 2019 (COVID-19) pandemic offer evidence of this dynamic. Despite data demonstrating that non-Hispanic Black populations and Hispanic populations were dying at a younger average age (71.8 years and 67.3 years) than non-Hispanic White patients were (80.9 years), the phase 1b vaccination strategy targeted individuals age 75 and older (25). Thus, federal public health recommendations ignored or discounted the evidence that an age-based approach would lead to further disparities in COVID-19 infections and mortality, amounting to structural racism against Black and Hispanic populations.

#### 2.3.2. The relationship between implicit bias and cognitive workload: overcrowding and patient load.

Studies have consistently shown that decision makers burdened with higher cognitive load are more likely to make biased decisions (10). A more recent study of physicians in the emergency department has confirmed that cognitive stressors such as patient overcrowding and patient load were associated with increased implicit racial bias as measured by a race IAT preshift compared to postshift (53).

#### 2.3.3. The relationship between implicit bias and the learning/training environment.

Unfortunately, to date, medical education and educators have not adequately addressed the implicit biases that place marginalized patients at high risk of receiving disparate care and suffering poorer health outcomes. In fact, Phelan et al. (84) concluded that structural racism is at play in medical education through many medical schools’ formal and hidden curricula (52, 88). In contrast to a formal curriculum, which can be measured by the number of hours students receive training related to racial disparities and bias, structured service-learning, minority health activities, cultural awareness programming, and the completion of an IAT, the hidden curriculum is unofficial and often more powerful, consisting of faculty role modeling (52), institutional priorities around the interracial climate, and experiences of microaggressions.

Most medical students continue to believe that both race and gender (as opposed to sex) are genetic and biological constructs. Even when students are taught otherwise, the practice of race-based medicine reinforces these characterizations. When students are taught about health disparities without the appropriate contextualization of structural racism, historic segregation, the pathologization of gender and sexual orientation, and the medical professions’ complicity in scientific racism, students may assume there is something inherently wrong with racialized minorities rather than with the systems that have harmed them. Students are often taught that race, instead of racism, is an independent risk factor for disease. They learn to associate race with any number of diseases. They are taught to incorporate the race of their patient into the opening line of clinical presentations even though there is no evidence that race is relevant to the establishment of diagnoses. They learn to use race-based algorithms to calculate glomerular filtration rates, pulmonary function testing, hypertension guidelines, and even urinary tract infection diagnoses in pediatric populations (2). Such messaging only serves to undo any structured teaching on the social construct of race and gender (16).

#### 2.3.4. The relationship between implicit bias and health care outcomes.

As discussed above, there is substantial evidence that implicit bias results in health care disparities through mechanisms including disparate care and trust. But the relationship between implicit bias and outcomes may be bidirectional. Evidence has shown that implicit attitudes are malleable and that such attitudes are learned and strengthened through repeated observation of particular classes of people in valued or devalued circumstances. For example, individuals exposed to less favorable exemplars from a given identity demonstrate increased implicit bias and stereotypes with respect to that entire group (20). Furthermore, these investigators showed that changing exposure to more favorable exemplars can diminish established implicit bias. This phenomenon has been demonstrated in experiments looking specifically at race- and age-related attitudes (21). These findings suggest that a practitioner’s implicit bias toward a marginalized group may be augmented or diminished by the clinical outcomes of that group.

#### 2.3.5. Favorable relationships between structural elements of training and bias: curricula, climate, and contact.

The CHANGES study demonstrated that students’ implicit bias against sexual minorities was reduced at 42 medical schools and increased at only 7 schools. Reduced bias was associated with more frequent interaction with LGBT students, faculty, and patients; the perceived quality of that contact; and increased training involving skills in caring for sexual minorities (85).

The CHANGES study found that changes in student implicit racial attitudes were independently associated with formal curricula related to disparities in health and health care, cultural competence, and minority health; informal curricula (or hidden curricula, defined in Table 1), including racial climate and role model behavior; and the amount and favorability of interracial contact during medical school (84).

Thus, carefully designed structural elements of the learning environment can favorably affect the implicit biases and wellness of students.

### 2.4. Systematic Review of Studies with Interventions

A systematic literature review was performed with the goal of assessing the efficacy of extant interventions designed to reduce the explicit and implicit biases of health care providers and of learners across the continuum of health professions education.

#### 2.4.1. Methods.

**Figure 1:**
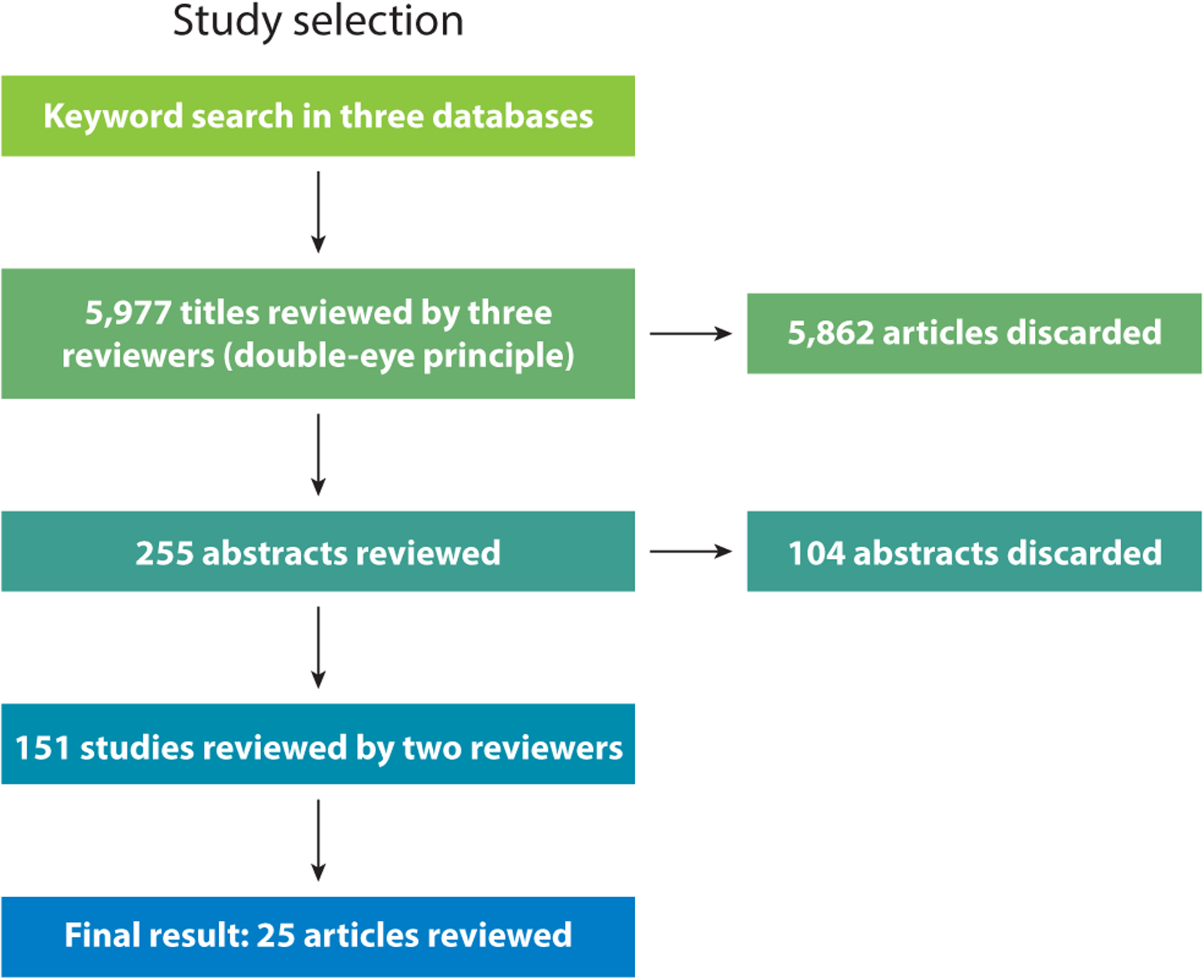
PRISMA flow diagram of the systematic review.

We searched three databases (ERIC, PubMed, and MedEdPORTAL) using key terms (Figure 1). The terms “implicit bias,” prejudice,” and “stigma” were often used inter-changeably and the terms “bias” and “biases” yielded more than 100,000 articles, often with little relevance to implicit bias in the health professions. We found, as did FitzGerald et al. (30) in their systematic review, that indexing in databases for these terms was inconsistent and that titles and abstracts were often imprecise. We conducted repeated searches with and without these terms, comparing the number of search results. We developed a set of terms most frequently encountered in the titles and abstracts of irrelevant articles and defined important terminology (Table 1) to narrow the search. We reviewed the references of landmark articles and used the advanced search function to increase the likelihood that no key articles were missed.

A study had to include health care professionals, assess an intervention (e.g., training, workshop, didactics, contact, program) designed to address explicit or implicit bias held by health care providers, be written in English, and be published between May 2011 and May 2021. We excluded commentaries, theoretical frameworks, editorials, and institutional or societal pledges that address racism, although these were reviewed for context. We did not exclude qualitative studies, studies without comparison groups, or studies outside North America. However, although we did find studies from other countries detailing explicit and implicit biases, we did not find articles with interventions addressing these biases for inclusion in this review. We extracted subjects, intervention format (e.g., lectures, workshops, discussions, panels, interviews), target (e.g., knowledge, skills, attitudes, IAT), and summary of key findings.

We excluded abstracts that did not include original research or bias reduction as an expected outcome; that did not employ a discrete intervention or, like the CHANGES study, retrospectively identified effective interventions; or that studied populations other than health professions students, trainees, or providers. We excluded articles that focused on self-stigma (e.g., from a diagnosis of obesity, HIV, sexually transmitted infection, mental health) and community-based interventions, as they were not focused specifically on the bias of health professionals. Observational studies without discrete interventions were excluded but were reviewed in Section 1.

Title, abstract, and full-text review were conducted by three authors (M.B.V., A.I.E., and N.A.S.) and coded to consensus.

#### 2.4.2. Findings.

Twenty-five studies met inclusion criteria (Table 3). None of the studies mentioned in Sections 1 and 2 met inclusion criteria but were reviewed because of their significant contributions to the understanding of the interactions of implicit bias in learning and clinical settings. Most studies (68%) engaged medical students and utilized classroom or web-based interventions. Most studies did not have a control group (72%) and none used actual clinical settings. Three studies focused on interventions for implicit bias of faculty serving on admissions or search committees.

## 3. DURATION OF INTERVENTION EFFECT

The three studies of faculty serving on admissions or search committees reported increased awareness of biases, but none reported bias reduction or long-lasting impact.

Three studies followed subjects 3, 4, and 6 months post-intervention, but only one noted a lasting positive impact (96).

## 4. NOVEL INTERVENTION CONTENT

All studies addressing implicit bias among health care providers raised awareness of implicit bias through didactic instruction, discussions, workshops or other reflection-based techniques (e.g., service-learning, photovoice, contact-based interventions, theater reading; see Table 4), or an IAT or similar measure.

Despite the limitations noted in Section 2, the IAT continues to be widely utilized. The IAT and other measures (32) of implicit bias, stigma, and attitudes toward groups of persons were used among subjects to (*a*) demonstrate the existence of participant implicit biases, (*b*) act as a springboard to create cognitive dissonance for oral and/or written reflection and to practice bias management skills, and (*c*) evaluate interventions. Gonzalez et al. (37) found that using the IAT without priming on its results and without a follow-up debriefing led some subjects (22%) to question the validity of the measure and the existence of implicit biases, and therefore advised judicious use of the IAT and trained facilitators. Subjects who accepted the results of the IAT were not able to develop management strategies for those biases without dedicated instruction.

Despite having low explicit bias based on a self-reported survey, admissions committee members at The Ohio State University College of Medicine (14) had high levels of implicit preference for White versus Black students as measured by the Black-White IAT. Results were presented to committee members with strategies to reduce implicit bias. The following admissions cycle resulted in an increase in underrepresented minority matriculation from 17% to 20%, a change that was not statistically significant.

Seventy-six percent of studies (8, 13, 14, 23, 28, 35–38, 48, 51, 58, 59, 77, 82, 94, 96, 99, 109) instructed on structural determinants such as structural racism and/or historic oppression of groups so that subjects could explore explicit and implicit biases. All these studies demonstrated an increased awareness of bias, and subjects often voiced a willingness to address their biases. Four studies explored the use of contact with groups with identities such as LGBTQI (58, 59) and persons with mental illness (27, 77) with positive and negative results, respectively.

In recognition that biases may be immutable in the current health care context but can be managed, educators have used transformative learning theory (TLT) in concert with implicit bias management techniques. TLT transforms the individual’s existing paradigm by disrupting assumptions and then engaging in critical reflection and dialogue to interpret the disruptions (68). TLT may move learners to an “inclusive, self-reflective and integrative frame of reference” (100, p. 718). This paired approach has had early success. Sherman et al. (96) engaged both residents and faculty in transformative learning to address issues of race, racism, and Whiteness and created an environment for critical dialogue incorporating practical recommendations for addressing implicit bias in clinical practice. Focus groups 4 months later revealed that subjects noted increased awareness of their biases and sustained commitment to addressing racial bias, to challenging their own clinical decision making, and to engaging leadership in dialogue regarding bias.

Gonzalez et al. (38) describe implicit bias recognition and management (IBRM), a process that promotes conscious awareness of biases and fosters behavioral changes. IBRM supposes that biases are difficult to reduce and should therefore be managed. IBRM has helped medical students interrupt biases in learning and clinical settings. Wu et al. (109) paired IAT administration with training to improve skills in bias literacy, emotional regulation, and allyship (Table 4). Trainees practiced these skills in clinical vignettes and improved their confidence in addressing bias in real-world settings. All three studies created a brave space to explore biases and emphasized continued practice and development of skills.

These studies have multiple limitations. They often lacked control groups or used pre- and postcomparison designs. They had limited longitudinal follow-up and often were not performed in real-world clinical or learning environments. Many studies did not focus on targeted outcomes, and most did not access the continuum of learners in medical education such as practicing health care providers and leadership. Most interventions had a limited one-time delivery with no opportunity to measure a dose- or time-dependent effect.

## 5. DISCUSSION

Many of the interventions demonstrated successful promotion of awareness of implicit bias held among subjects as well as an interest in mitigating implicit biases among subjects. No intervention in this review, however, achieved sustained reduction of implicit bias among health care professionals or trainees. In addition, no study demonstrated that an intervention improved clinical outcomes, the learning environment, interprofessional team dynamics, patient care, health disparities, patient satisfaction, or satisfaction of health professionals. Studies were hampered by lack of statistical analysis, lack of control group, limited numbers of participants, findings that are not necessarily generalizable from the classroom or web-based setting to the clinical or real-world setting, and heavy reliance on qualitative assessments or nonvalidated instruments. Future studies should also assess whether regularly timed booster interventions manifest in sustained changes over time and should have longer-term follow-up to assess sustainability of initial gains. Future studies should include educational models that use direct clinical observation or standardized patients. Studies should assess health care trainees’ ability to incorporate skills into patient communication and shared decision making, their improvement of clinical delivery practices, their interactions with colleagues, and their teaching practices.

### 5.1. Conceptual Model

**Figure 2:**
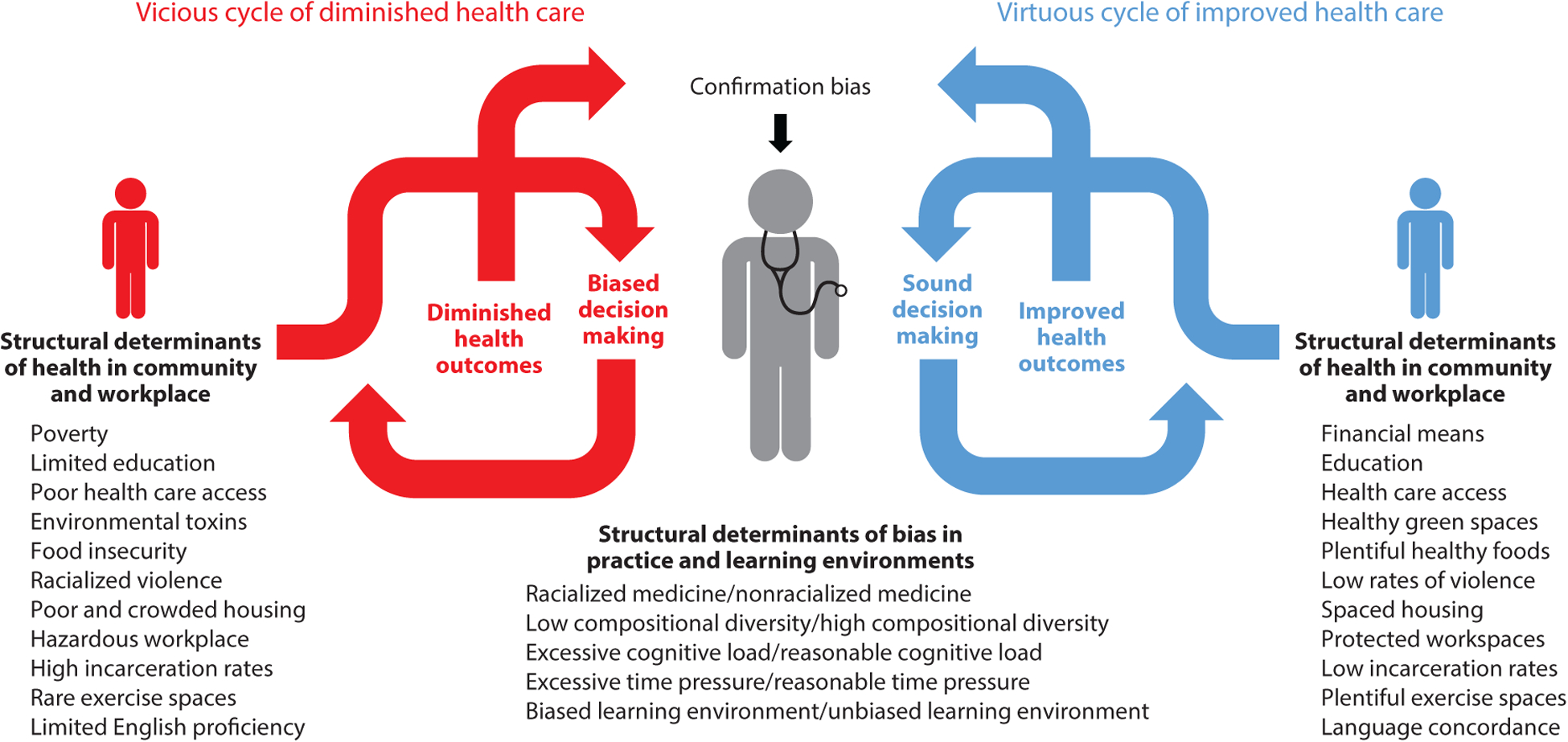
Interactions between structural determinants and provider implicit bias. The vicious cycle: Structural determinants of implicit bias in the practice environment support biased decision making. Structural determinants of health in the community further impair outcomes in marginalized populations, leading to confirmation of the practitioner’s implicit bias. Health disparities are exacerbated. The virtuous cycle: A favorable practice environment regarding structural determinants of implicit bias supports unbiased clinical decision making. Favorable structural determinants of health in the community further enhance patient outcomes, positively reinforcing unbiased practice. Health disparities are reduced.

Based on Jones’s (54) allegory *A Gardener’s Tale*, we present a conceptual model of implicit biases of health care providers and the key structural factors affecting these biases (Figure 2). In the vicious cycle of health disparity, students, trainees, and providers receive a constant barrage of messaging that reinforces biases. The soil of their work (practice and learning environments) is laden with structural bias from racialized medicine, a biased learning environment, and poor compositional diversity. Furthermore, these trainees and health care providers are under substantial time pressure and cognitive load. These characteristics of the practice and learning environments may be considered structural determinants of implicit bias.

Biases are now primed as the clinician moves to provide care to patients (see the left side of Figure 2). When caring for marginalized patients, the provider’s bias influences communication with the patient, potentially resulting in suboptimal decision making. The patient may sense the bias, may distrust the provider and system, and may decide to not follow through on treatment plans or may modify them. The patient lives in underresourced and unhealthy spaces that contribute to poor outcomes. The provider notes the poor outcomes and their implicit bias is confirmed. Health care disparities are exacerbated. Further exacerbation of the vicious cycle occurs when this dynamic is accompanied with biases toward students, trainees, and providers from marginalized groups. Individuals from these marginalized groups are less likely to succeed, confirming biases about them and perpetuating poor diversity in the health care workforce. The benefits of diversity to education and patient care are lost.

The right side of Figure 2 depicts the virtuous cycle of health equity. A well-resourced provider learning and working within an environment devoid of racialized medicine and bias and characterized by compositional diversity is less likely to display biases against the patient. Compositional diversity also increases the likelihood that the provider shares lived experiences with the patient. The patient notes the absence of provider bias, develops a trusting relationship, adheres to the treatment plan in a well-resourced environment, and returns with improved health outcomes. The patient’s outcome confirms the provider’s more favorable bias. Health care disparities are reduced.

This conceptual model highlights two important dynamics in the perpetuation of implicit bias and its impact on care. First, structural determinants in the health care system and surrounding community contribute to the development of implicit bias toward marginalized patient populations and then reinforce that implicit bias through generation of poorer patient outcomes. Second, interruption of this cycle is possible only through an overall shift toward favorable structural influences on implicit bias. Discrete, time-limited training as the sole intervention to reduce implicit bias is unlikely to result in sustained change; health care providers return to a practice or learning environment that is often replete with structural determinants and patient outcomes that reinforce implicit bias. To avoid the ongoing creation and perpetuation of racist structures in society, systems, and organizations, it is crucial to recognize that these dynamics may enhance the implicit bias of medical leaders and policy makers as well.

### 5.2. Taking Action

To enable provider-level bias interventions to succeed in improving health outcomes, multiple other concurrent approaches should address structural factors inside and outside the health care system that influence these biases (80).

Structural inequities outside the health care system include poor access to high-quality health care, racialized violence, the carceral state, crowded housing, healthy food scarcity, lack of access to green spaces, environmental toxins, and poorly protected workspaces, among other issues related to geography and place (19, 103).

Structural inequities inside the health care system that prime bias include the work and learning environments of students, trainees, and providers (104). It will be important to address these structural drivers of bias, including time pressures, cognitive load, and the practice of racialized medicine. Racism, sex and gender discrimination, and other forms of discrimination must be rooted out, as they prevent marginalized trainees and faculty from thriving, create stereotype threat for the marginalized, and confirm bias for the nonmarginalized. Bioethical principles of fairness, distributive justice, and reciprocity should be core for public health officials and health care providers, and practitioner and provider trainings in these areas can raise awareness. For example, to address health inequities laid bare by COVID-19, Peek et al. (79) recommend a multifactorial approach that acknowledges the systemic racism of the health care system and other societal structures as well as the biases of providers (67).

Addressing compositional diversity in health care is another avenue for treating the structures that influence implicit and explicit biases and eliminate health care disparities. Minority health professionals are underrepresented in the workforce and health professions faculty (60). Only 6.2% of medical students identify as Hispanic or Latinx, and only 8.4% as Black or African American (1). Gender parity among medical school students has been achieved. However, women are underrepresented at the faculty instructor level, with substantially less representation at the professor level, and are also underrepresented in hospital leadership, with even starker inequities for female racial and ethnic minorities (33, 88). Gender inequalities in salaries have been well documented (12, 62, 71). In academic medicine, Black male faculty are offered lower rates of compensation than their White counterparts and are less likely to be awarded research funding from the National Institutes of Health (34). Similarly, in 2016, graduate student enrollment in the Association of Schools and Programs of Public Health demonstrated a ≤5% increase over a 20-year period among Asian, Black, Hispanic, and Native American students; only 11.1% of students were Black and 12% were Hispanic. Black, Hispanic, and Native American representation among tenured public health faculty increased <3% during this same 20-year period (39).

## 6. CONCLUSION

TLT, IBRM, and a skills-based approach offer promise for future interventions in implicit bias management. It is also encouraging that discussions around disparities and inequities have moved from race to racism and have focused on the professional responsibility of providers to root out inequities and manage biases. The extant literature regarding the use of provider-level implicit bias interventions suggests that these interventions can play an important role in concert with other interventions that more broadly address bias and discrimination inside and outside the health care system. Evidence supports the use of provider-level interventions in immediate-impact activities such as decision making on search committees or admissions committees and raising critical awareness of the bioethical principles of fairness, distributive justice, and reciprocity. However, provider-level implicit bias interventions alone have not improved health outcomes. Thus, provider-level implicit bias interventions should be accompanied by interventions that systemically change structures inside and outside the health care system that influence biases and perpetuate health inequities.

## Figures and Tables

**Table 1:** Terminology of bias

Term	Definition
Discrimination	Discrimination is “the result of either implicit or explicit biases and is the inequitable treatment and/or impact of general policies, practices, and norms on individuals and communities based on social group membership” (64, p. S5).
Ethnicity	Ethnicity is “a social system defining a group that shares a common ancestry, history or culture with some combination of shared geographic origins, family patterns, language, or cultural norms, religious traditions, or other cultural and social characteristics” (106, p. 325).
Explicit bias	Explicit forms of bias include “preferences, beliefs, and attitudes of which people are generally consciously aware, endorsed, and can be identified and communicated” (22, p. 1).
Hidden curriculum	“Lessons taught through socialization of learners especially as it pertains to professionalism, humanism, and accountability, as opposed to explicitly taught in the classroom or bedside” (89, p. 50).
Implicit bias	Implicit biases are “unconscious mental processes that lead to associations and reactions that are automatic and without intention and actors have no awareness of the associations with a stimulus. Implicit bias goes beyond stereotyping to include favorable or unfavorable evaluations toward groups of people.” While we are not aware these implicit biases exist, they have a significant impact on decision making (97, p. 14).
Institutional racism	Institutional racism (structural) “refers to the processes of racism that are embedded in laws (local, state and federal), policies, and practices of society and its institutions that provide advantages to racial groups deemed superior while differentially oppressing, disadvantaging or otherwise neglecting racial groups viewed as inferior” (107, p. 107).
Race	“Race is primarily a social category, based on nationality, ethnicity, phenotypic or other markers of social difference, which captures differential access to power and resources in society. It functions on many levels and socializes people to accept as true the inferiority of nondominant racial groups leading to negative normative beliefs (stereotypes) and attitudes (prejudice) toward stigmatized racial groups which undergird differential treatment of members of these groups by both individuals and social institutions” (107, p. 106).
Racism	“Racism is an organized social system in which the dominant racial group, based on an ideology of inferiority, categorizes and ranks people into social groups called ‘races’ and uses its power to devalue, disempower, and differentially allocate valued society resources and opportunities to groups defined as inferior... A characteristic of racism is that its structure and ideology can persist in governmental and institutional policies in the absence of individual actors who are explicitly racially prejudiced” (107, p. 106).
Role modeling	Role modeling is a mechanism for teaching behavior through learning by observation (52, p. 26).
Stereotype	A stereotype is “a fixed set of attributes associated with a social group” (49, p. 209).
Stereotype threat	Stereotype threat “occurs when cues in the environment make negative stereotypes associated with an individual’s group status salient, triggering physiological and psychological processes that have detrimental consequences for behavior” and performance of the individual who identifies as a member of the stereotyped group (11, p. S169).

**Table 2:** Impacts of implicit bias

Area	Impacts
Health care delivery	Patient-provider communication Patient-provider relationships Patient satisfaction Patient perception of physician’s patient-centeredness Patient treatment adherence Provider decision making Provider’s perspective of patient’s likelihood to adhere to treatment
Public health	Resource allocation (testing locations, vaccine distribution, location of environmental stressors)
Health professions workplace and learning environments	Promotions practices Compensation Evaluations Awards and recognition Research grants Stress, isolation
Diversity of trainees and workforce	Recruitment and selection of future trainees Inclusive learning environment

**Table 3:** Provider-level implicit bias interventions

	Study population	Intervention	Evaluation/outcomes	Limitations	Reference
Interventions without formal measurement of implicit bias/attitudes	Medical students (*n* = 25)	Study and control groups Study group participated in 5-h dialogues on race and bias	Pre- and postsurveys Paired *t*-tests demonstrated increased knowledge and awareness of racial bias and increased comfort talking about race.	No formal bias measure Self-selected study group of students	8
	Faculty who serve on search committees (*n* = 22)	2-h reflection-based workshop on unconscious bias	Post-intervention survey evaluated effectiveness and utility of exercise. Most surveyed found workshop helpful in preparing for faculty searches.	Extremely limited evaluation (no pre-/postcomparison) No formal bias measure	13
	Medical students *(n* = 615)	2-day orientation on power, privilege, and bias	Post-intervention survey Surveys demonstrated raised bias awareness.	No formal bias measure No pre-/postcomparison	23
	Medical students (*n* = 187)	Five 2-h workshops with lectures on bias	Pre- and postsurveys Paired *t*-tests on surveys demonstrated raised awareness of own biases and intent to address bias.	No formal bias measure	28
	Health professions educators *(n* = 70)	Introduced new longitudinal case conference curriculum called HER to discuss and address the impact of structural racism and implicit bias on patient care Utilized case-based discussion, evidence-based exercises, and two conceptual frameworks	Tracked conference attendance and postconference surveys Most survey respondents (88% or more) indicated that HER promoted personal reflection on implicit bias, and 7 5 % or more indicated that HER would affect their clinical practice.	No pre-/postcomparison No formal bias measure No control group	82
	Faculty *(n* = 66)	90-min interactive workshop that included a reflective exercise, role-play, brief didactic session, and case-based discussion on use of language in patient charts	Post-intervention survey with four Likert scale questions Participants felt workshop met its objectives (4.8 out of 5.0) and strongly agreed that they would apply skills learned (4.8).	Self-selected study group No measure of bias No control group	90
	Family medicine residents (*n* = 31)	Training on institutional racism, colonization, and cultural power followed by humanism and instruction on taking health equity time-outs during clinical time	Focus groups conducted 6 months post-intervention Four themes: increased awareness of and commitment to addressing racial bias appreciation of a safe forum for sharing concerns new ways of addressing and managing bias (i.e., challenged their clinical decision making) institutional capacity building for continued vigilance and training regarding implicit bias	No measure of bias No pre-/postcomparison Qualitative analysis only No control group	96
	Medical students (*n* = 26)	Service-learning plus reflection	Reflection practice questionnaire analysis Students reported recognizing and mitigating bias.	No formal measure of bias used No control group	51
	Medical students (*n* = 127)	Readings/reflections on weight stigma Standardized patient before and after	Pre-/post-intervention questionnaires Reduced stereotyping, increased empathy, and improved counseling confidence Weak analysis may be biased itself.	No formal bias measurement No control group	56
Interventions with formal measurement of implicit bias/attitudes	Medical students/elective (*n* = 218)	Single session in which students completed an IAT followed by discussion	Post IAT survey Implicit bias deniers were significantly more likely to report IAT results with implicit preferences toward self, to believe the IAT is invalid, and to believe that doctors and the health care system provide equal care to all, and were less likely to report having directly observed inequitable care.	Self-selected study group No control group	36
	Medical students (*n* = 180)	Single IAT administration followed by guided reflective discussion and essay writing	Evaluation of reflective essays Students noted raised awareness of bias but were not able to strategize solutions to mitigate bias.	Prompt did not ask for strategies No control group	37
	Medical students (*n* = 15)	Nine 1.5-h sessions focused on promoting skills to empower students to recognize implicit bias reduction as part of professionalism Three objectives (grounded in implicit bias recognition and transformative learning theory): recognize when implicit bias may be influencing one’s own communication with a patient or peer through reflection and by taking an IAT advocate on behalf of patients when perceiving bias in a witnessed encounter address biased comments made within the learning environment	Post-intervention focus groups and analysis of semistructured interviews Major themes: student engagement can be enhanced instruction is empowering addressing bias in one’s own and witnessed encounters is feasible	Self-selected small group of students No control group	38
	Medical students (*n* = 72)	IAT administration followed by small group debrief and discussion on bias	Qualitative analysis of discussion transcripts Students who reach for normative versus personal standards had higher implicit bias post-intervention.	No post IAT measure of bias No control group	48
	Nursing students (*n* = 75)	Pre/post IAT with debriefing, writing, and teaching of bias management techniques (e.g., internal feedback, humanism)	Postclass survey, conducted 5 weeks after the intervention Learners were extremely likely or likely to (*a*) take additional IATs and reflect on the results and (*b*) learn more about unconscious bias.	No formal analysis of pre/post IATs, but focus was on acceptance of bias and management No control group	94
	Medical students (*n* = 78)	Workshops that involved IAT administration, instruction on implicit bias and impact on decision making, and presentation of six strategies to reduce implicit bias	Reduction of implicit bias against Hispanics as measured by an IAT in majority students only No change for minority students was demonstrated.	No control group Nonclinical setting	99
	Medical students, house staff, faculty (*n* = 468)	Twenty workshops to emphasize skill building and include lectures, guided reflections, and facilitated discussions focused on the following: an overview of unconscious bias which involves IAT administration followed by skills on bias literacy and emotional regulation an introduction to allyship vignettes, in which participants use cases to practice skills introduced in the previous sections	Survey response rate was 80%; a paired *t*-test Pre- and postsurveys to evaluate the intervention’s capacity to improve awareness of bias and address it through allyship Demonstrated greatest improvements in understanding of the process of allyship; ability to describe strategies to address, assess, and recognize unconscious bias; and knowledge of managing situations in which prejudice, power, and privilege are involved	Improved confidence in addressing bias but no measure of bias reduction	109
	Faculty on admissions committee (*n* = 140)	Black-White IAT administered before 2012–2013 medical school admission cycle Study participants received results before start of admission cycle and were surveyed on the impact at the end of cycle in May 2013	Most survey respondents (67%) thought the IAT might be helpful in reducing bias, 48% were conscious of their individual results when interviewing candidates in the next cycle, and 21 % reported knowledge of their IAT results impacted their admissions decisions in the subsequent cycle. This class is the most diverse to matriculate in the Ohio State University College of Medicine’s history.	Unclear whether other factors affected matriculation of students	14
	Faculty members (*n* = 281)	Standardized, 20-min educational intervention to educate faculty about implicit biases and strategies for overcoming them	Pre-/postassessments that included the following: a survey measuring general perceptions of bias an assessment of measures of explicit attitudes related to gender and leadership a version of the IAT measuring the association between gender and leadership The intervention had a small but significant effect on the implicit biases surrounding women and leadership of all participants regardless of age and gender. Faculty experienced significant increases in their perceptions of personal bias (Cohen’s *d* = 0.50 and 0.17; *p* < 0.01 for both questions), perceptions of societal bias (Cohen’s *d* = 0.14, 0.12, and 0.25; *p* < 0.05 for all three questions), and perceptions of bias in academic medicine (Cohen’s *d* = 0.38, 0.57, and 0.58; *p* < 0.001 for all three questions).	Immediate impact only No control group	35
	Medical students (*n* = 64)	Study participants watched video linking obesity to genetics and environment	Beliefs about Obese Persons, Attitudes toward Obese Persons, and Fat Phobia Scales administered pre- and post-intervention Paired *t*-tests revealed decreased negative stereotypes and beliefs.	No longitudinal results No control group	87
	House staff (*n* = 69)	Narrative photography to prompt reflection and photovoice of Latino adolescents	Control and intervention groups Measured ethnocultural empathy, health care empathy, patient centeredness, and implicit attitudes using the affect misattribution procedure All measures improved with some note of dose response with more exposure.	Nonclinical setting	17
	Medical students (*n* = 129)	Workshop to address obesity-related bias using theater reading (intervention group) of play versus lecture (control group) on obesity Students randomly assigned to groups	Obesity-specific IAT, anti-fat attitudes questionnaire pre-/postworkshop Reduced explicit fat bias in theater group with no change in implicit bias or empathy post-intervention or 4 months later	Nonclinical setting	66
	Primary care providers (*n* = 185)	Study participants randomized to intervention (lecture and contact)/control (lecture and discussion)	Beliefs and Attitudes towards Mental Health Service Users’ Rights Scale Reduced stigmatizing beliefs and attitudes at 1 month in the intervention group but rebound effect at 3 months	No formal measure of bias Nonclinical setting	27
	Medical students (*n* = 111)	One-time contact-based educational intervention on the stigma of mental illness among medical students and compared this with a multimodal undergraduate psychiatry course	Opening Minds Scale for Health Care Providers to assess changes in stigma Stigma scores for both groups were significandy reduced upon course completion (*p* < 0.0001) but were not significandy changed following the one-time contact-based educational intervention in the primary analysis.	Nonclinical setting	77
	Medical students (*n* = 160)	Intergroup contact theory (facilitated contact to reduce bias) plus 50 h of competency-based curriculum on inclusive care of LGBTQ and gender-nonconforming individuals through lectures, standardized patients, discussion, panels, and reflective writing	Had study and control groups Pre and post IATs with debriefings demonstrated reduced implicit preference for straight people. IAT with debriefings were important when used to facilitate curriculum.	Nonclinical setting	59
	Medical students (*n* = 50)	Three cultural competency training sessions led by LGBTQ2S+ experts and elders from the community Study participants randomized to intervention and control groups Focus group discussions conducted	Pre-/postassessment Lesbian, Gay, and Bisexual Knowledge and Attitudes Scale for Heterosexuals and The Riddle Scale: Attitudes towards Gay, Lesbian, Bisexual, and Trans people survey Measurable and relevant changes in health care students’ perceived knowledge, attitudes, and clinical behavior regarding LGBTQ2S+ populations as a result	Nonclinical setting	58

Abbreviations: HER, Health Equity Round; IAT, implicit association test.

**Table 4:** Definitions of intervention types used in selected studies

Intervention type	Definition
Allyship training	“An active, consistent, and arduous practice of unlearning and re-evaluating, in which a person of privilege seeks to operate in solidarity with a marginalized group” (72) “Allyship begins with an awareness of unconscious biases and then moves to actions that address inequities in everyday interactions to create an inclusive culture for example to amplify the voices of those in underrepresented groups and to advocate for equitable practices” (33, p. 6).
Bias literacy	Promotes a basic understanding of key terms, skills and concepts related to bias as a first step to organizational change (15, p. 64; 95, p. 22)
Brave space	“A space where difficult, diverse, and often controversial issues are presented and can be discussed with a common goal of understanding the barriers to equity in health care” (92, p. 87)
Emotional regulation	“The processes by which we influence which emotions we have, when we have them, and how we experience and express them” (46, p. 282)
Intergroup contact	The promotion of contact between two groups with the goal of reducing prejudice (83, p. 66)
Photovoice	“A method that allows participants to use photography to document their experiences and dialogue to eventually influence change” (61, p. 318)
Service-learning	A “pedagogy of engagement wherein students address a genuine community need by engaging in volunteer service that is connected explicitly to the academic curriculum through structured ongoing reflections” (98, p. 115)
Theater reading	Play reading with students as active participants (66, p. 232)
